# Transformation of *Epichloë typhina *by electroporation of conidia

**DOI:** 10.1186/1756-0500-4-46

**Published:** 2011-03-05

**Authors:** James E Dombrowski, James C Baldwin, Steve C Alderman, Ruth C Martin

**Affiliations:** 1 USDA-ARS National Forage Seed Production Research Center, 3450 S.W. Campus Way, Corvallis, OR 97331 USA; 2 Applied Technology Center, 2484 Gillingham Drive, B-175W Brooks City-Base, TX 78235 USA

## Abstract

**Background:**

Choke, caused by the endophytic fungus *Epichloë typhina*, is an important disease affecting orchardgrass (*Dactylis glomerata *L.) seed production in the Willamette Valley. Little is known concerning the conditions necessary for successful infection of orchardgrass by *E. typhina*. Detection of *E. typhina *in plants early in the disease cycle can be difficult due to the sparse distribution of hyphae in the plant. Therefore, a sensitive method to detect fungal infection in plants would provide an invaluable tool for elucidating the conditions for establishment of infection in orchardgrass. Utilization of a marker gene, such as the green fluorescent protein (GFP), transformed into *Epichloë *will facilitate characterization of the initial stages of infection and establishment of the fungus in plants.

**Findings:**

We have developed a rapid, efficient, and reproducible transformation method using electroporation of germinating *Epichloë *conidia isolated from infected plants.

**Conclusions:**

The GFP labelled *E. typhina *provides a valuable molecular tool to researchers studying conditions and mechanisms involved in the establishment of choke disease in orchardgrass.

## Introduction

Orchardgrass (*Dactylis glomerata *L.) is an important forage grass species. About 97% of orchardgrass seed used for pastures and hay in North America is produced in the Willamette Valley in western Oregon. Choke disease, caused by the endophytic fungus *Epichloë typhina*, was first reported in the Willamette Valley during the mid 1990s [1]. Infected plants remain asymptomatic during most of the year. In the spring, *E. typhina *proliferates within reproductive tillers, encasing the developing seed head in a dense mycelial mat (stroma). However, the stem continues to grow, revealing an elongated, white stromal mass that resembles a small cattail. Conidia of one or two mating types are produced on the surface of each stroma. Fertilization requires the transfer of conidia of one mating type to stroma of the opposite mating type. This is typically accomplished by flies in the genus *Botanophila *[2]. Following fertilization, perithecia develop within the stroma surface [3,4]. The perithecia produce ascospores, which are dispersed by wind and cause new plant infections. The pathogen has not been shown to be transmitted through the seed in orchardgrass, but it is seed transmitted in other grasses [3-6].

The recent introduction and rapid spread of choke disease is a serious problem for orchardgrass seed producers in the Willamette Valley [1,7,8]. In England, where the disease has occurred for many years, the number of fields with choke disease increased from zero to a few during the first year of production to 33-81% by the second to fifth year of seed production [9]. In France, choke was reported to affect up to 30% of the tillers in a field by the fourth year of seed production [10,11]. Since the first incidence of choke reported in Oregon in the mid 1990s [1], the fungus has spread to ~90% of orchardgrass seed production fields and has caused yield losses of up to 65% in individual fields [8].

Very little is known about the conditions necessary for successful infection of orchardgrass by *E. typhina*. Fertilization of the fungal stroma is facilitated by flies [2,12], but fly density was not correlated with reproductive success of the fungus in western Oregon [2,13]. Fertilization of *E. typhina *by ascospores has been reported recently and may contribute to the rapid spread of choke in orchardgrass [14]. Attempts to infect orchardgrass foliage or flowers with conidia or ascospores were not successful [4]. Germination of *E. typhina *ascospores and conidia on the cut ends of seed stalks of orchardgrass and growth of hyphae down the pith has been reported [4-6], although it was not established whether these plants ultimately became infected through the stalks. Infection of young tillers by ascospores or conidia produced by ascospores under very favorable experimental conditions was recently reported [15]. It is not known when or how infections occur in the field or how long the latent period is between infection and manifestation of symptoms in the field.

Detection of *E. typhina *early in the disease cycle can be difficult due to sparse distribution of hyphae in the plant. Therefore a sensitive method to detect the fungus in plants would provide an invaluable tool for elucidating the conditions and establishment of the infection in orchardgrass. Green Fluorescent Protein (GFP) has been utilized extensively as a marker to aid in the development of fungal transformation systems and to examine early stages in plant/fungal interactions [reviewed in [16],[17-22]]. Translation fusions between *GFP *and fungal genes of interest have also been utilized to determine gene expression patterns and to investigate the role of specific genes in the infection process [16,23]. Transformation of *E. typhina *with the GFP marker gene will facilitate the characterization of the initial stages of infection and progression of the disease in plants [16]. There are several different methods available for fungal transformation, such as chemical induction, *Agrobacterium*-mediated transformation, particle bombardment and electroporation [[16,24-27], reviews and references within, [28,29]]. Chemical transformation of protoplasts of *Epichloë festucae *has been reported and was used to determine the expression patterns of the lolitrem biosynthetic genes [30] and to look at the infection process of *E. festucae *in *Lolium perenne *[31]. Electroporation of germinating conidia has been used successfully to transform a range of fungal species [32-34]. We report here a simple and rapid method using electroporation to transform germinating *Epichloë *conidia isolated from infected plants. The GFP labelled *E. typhina *provides a valuable molecular tool for researchers studying the infection process in orchardgrass. The transformation protocol will also be valuable for gene function studies in *Epichloë*-plant interactions.

## Materials and methods

### Transformation vector

The pCT74 GFP expression vector [16] was used as an intact circular plasmid or as a linearized plasmid for transformation experiments. The plasmid was digested overnight at 37°C with Xho I to linearize the plasmid. The digested DNA was precipitated with 1/10 volume of 3 M NaOAc, pH 5.2, and 2 volumes of EtOH. One μL of blue dextran (10 mg/mL) was added to aid in visualization of the pellet. The precipitated DNA was resuspended in sterile distilled water at a final concentration of 0.5 μg/μL.

### Collection and preparation of conidia for transformation

**Figure 1:**
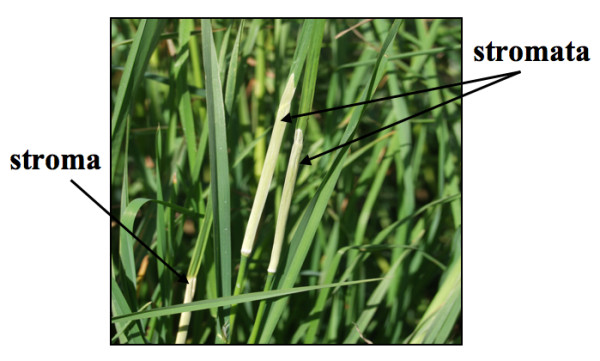
***Epichloë typhina *infection of orchardgrass**. Conidia are collected from the whitish, felt-like stroma that develops on reproductive tillers as they elongate, just prior to seed head emergence.

Orchardgrass plants known to be infected with *E. typhina *were grown in the greenhouse under 16 hr of light at 21°C. To induce flowering, the plants were vernalized in a growth chamber at 8°C with 8 hr of light for 12 weeks, then at 15°C for 2 additional weeks under 18 hr of light, and then returned to the greenhouse. Conidia from newly formed unfertilized stromata (Figure 1) were collected in about 10 mL of sterile water using a sterile artist paintbrush. The brush was dipped into sterile deionized water, gently brushed along the surface of each stroma to collect conidia in the brush, then shaken in the water to dislodge the spores. Approximately 10^6 ^conidia/mL were obtained when conidia from 5-10 stroma were collected in one tube. Isolated conidia were gently shaken in sterile deionized water for 4 hr at room temperature at which time >90% of the conidia had germinated (Additional File 1). Suspended germinated conidia were transferred into sterile microfuge tubes (1.5 mL/tube) and centrifuged at ~16,000 × g for 45-60 s. Approximately 1.45 mL of the supernatant was discarded. The conidia were resuspended in cold sterile electroporation buffer (1 mM Hepes, 50 mM mannitol, pH 7.5) to a final volume of 1 mL.

### Sensitivity of untransformed conidia to hygromycin B

The plasmid used for transformation contains the hygromycin B phosphotransferase gene which confers resistance to hygromycin and enables the selection of transformed colonies. To determine the concentration of hygromycin to be used for selection, conidia (~5000 conidia/plate) were placed on increasing concentrations of hygromycin B, from 0-400 mg/L, on Corn Meal Malt Agar (CMMA - 19 g corn meal agar, 2 g yeast extract, 20 g malt extract and 5 g agar per liter). Since conidia were collected from plants grown in the greenhouse, 150 μg/mL of ampicillin or ticarcillin was added to the selection medium to inhibit bacterial growth.

### Determination of optimal electrical field strength for conidia transformation

Electroporation was performed using the Bio-Rad Gene Pulser II on 100 μL of suspended conidia (~5000 conidia) in a 0.2 cm cuvette. The percent survival of germinating conidia at various combinations of voltage settings between 1 and 2 kV, resistances between 400-800 ohms, and 25 μF capacitance was tested. Percent survival at each resistance level (400, 600, 800 ohms) at the three voltages was determined by calculating the number of colonies from each sample (based on colony counts obtained from plating out serial dilutions from each sample) and comparing that to the number of colonies obtained from non-electroporated germinating conidia.

### Transformation of conidia

Approximately 10^5^ — 10^6 ^conidia in 100 μL of sterile electroporation buffer were combined with 0.1-2.0 μg of circular or linearized DNA in sterile 500 μL microfuge tubes and incubated on ice for 20 min. The DNA/conidial mixture was then transferred into a cold 0.2 cm electroporation cuvette and subjected to a specific electrical field using Bio-Rad Gene Pulser II. Immediately following the pulse, the conidia were transferred to a sterile 15 mL snap-cap tube containing 900 μL of cold sterile Regeneration Medium (RM - 14.5 g mannitol, 0.4 g yeast extract and 1.5 g potato dextrose broth per 100 mL distilled water). The conidia were incubated on ice for 20-30 min and were then placed on a rotating shaker (150-180 rpm) at room temperature for 5 hours prior to plating on selective solid medium (200 μL/plate). Since conidia were collected from plants grown in the greenhouse, 150 μg/mL of ampicillin or ticarcillin was added to the selection medium to inhibit bacterial growth. Hygromycin B-resistant colonies started to develop in 3-4 weeks. Colonies were examined with a LEICA Fluorescent Dissecting Microscope MZFLIII using a PLANAPO 1.0X Lens to confirm GFP expression in *E. typhina *transformants. In order to visualize the untransformed control fungus, low intensity white light was used in addition to the UV light.

### Southern analysis to determine copy number

DNA was isolated using a modified CTAB (hexadecyltrimethylammonium bromide) protocol [35] including treatment with RNase. The DNA was treated with Nucleon PhytoPure™resin, a component found in the Illustra Nucleon Phytopure™Genomic DNA extraction kit (GE Healthcare UK Limited, Buckinghamshire, UK) which covalently binds polysaccharides. Two μL of the resin was added per 100 μL of solution, mixed gently, and then centrifuged to pellet the resin. The DNA remained in the supernatant. Approximately 5 μg of DNA was digested with EcoRI, using the manufacturer's supplied buffer, and separated in a 1.2% agarose gel. DNA was transferred to a Hybond-N+ membrane (Amersham; GE Healthcare) by downward capillary transfer following standard protocols [36]. PCR was used to synthesize a digoxigenin (DIG; Roche, Cat. No. 11573152910) labelled probe for a portion of the GFP gene following the recommendations of the company (https://www.roche-applied-science.com). Primers were designed using Primer3 software [37]. Primers for probe synthesis were GFPF64 5'-GACGTAAACGGCCACAAGTTC and GFPR671 5'-GAACTCCAGCAGGACCATGTG producing an amplicon of ~ 600 bp (between nucleotide 64 and 671 of the *GFP *open reading frame). Hybridization and detection were performed following the protocol of Engler-Bloom et al. [38] with modifications described by Krueger and Williams [39].

## Results and discussion

### Sensitivity of untransformed conidia to hygromycin B

**Figure 2:**
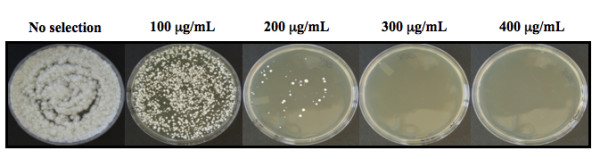
**Sensitivity of *Epichloë typhina *conidia to hygromycin B**. Selection of untransformed conidia (5000 conidia/plate) on increasing concentrations of hygromycin B on Corn Meal Malt Agar (CMMA). As shown above, *E. typhina *conidia demonstrated resistance to hygromycim B. Based on this analysis we decided to use 200 μg/mL and 300 μg/mL of hygromycin B for selection of transformants.

When conidia (~5000 conidia/plate) were placed on increasing concentrations of hygromycin B on CMMA, many colonies were present on 0 and 100 μg/mL hygromycin, but only a few colonies grew at 200 μg/mL, and no colonies grew at 300 and 400 μg/mL hygromycin (Figure 2). Based on this analysis, hygromycin B concentrations of 200 and 300 μg/mL were used for selection of transformants.

### Determination of optimal electrical field strength for conidia transformation

Most reports indicate that the best electroporation conditions to use for transformation are those that result in 40-60% survival of the cells. With increasing levels of resistance, the 40-60% survival rate occurred at lower voltages. Approximately 50% of the conidia survived using 1 kV at 800 Ω, 1.2 kV at 600 Ω, and between 1.2 and 1.5 kV at 400 Ω (Additional file 2).

### Transformation of conidia

**Figure 3:**
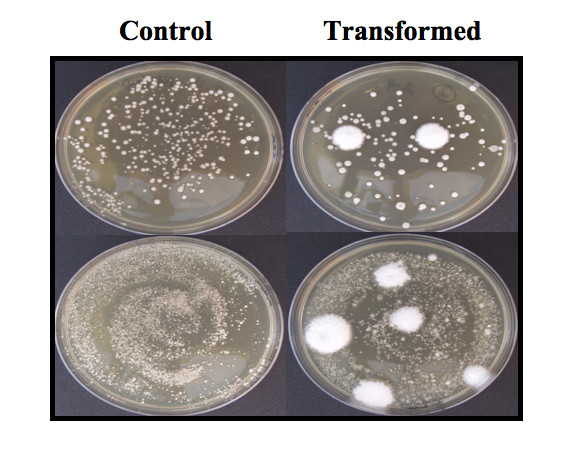
**Transformants of *Epichloë typhina***. Resistant colonies (large) growing on 200 μg/mL hygromycin B CMMA plates (right) and background colonies on control plates (left).

Hygromycin B-resistant colonies started to develop in 3-4 weeks. Transformed colonies grew to a much larger size than untransformed background colonies (Figure 3). Transformation efficiency was greatly affected by use of an intact versus linearized vector and the age of the stroma. The linearized plasmid resulted in a much higher frequency of transformation than the circular plasmid which yielded only two transformants. It was also important that conidia were collected from newly formed stroma, as conidia isolated from more mature stroma were not transformable. The greatest number of transformants was obtained at 400 Ω resistance and 1.25 kV at a capacitance of 25 μF. At resistances of 600 and 800 Ω, a voltage of 1.0 kV gave a higher number of transformants compared to what was obtained at 1.25 kV and 1.5 kV (Table 1).

**Table 1:** Number of hygromycin-resistant colonies expressing GFP at designated electroporation conditions.

		# GFP/total # Hyg^R ^colonies				Overall	
		
Ω	kV	1	2	3	4	GFP/Hyg	%GFP
**400**	1	26/28	23/35	18/24	20/26	87/113	77

**400**	1.25	36/45	28/37	26/39	41/48	131/169	78

**400**	1.5	15/19	18/21	17/20	27/32	77/92	84

**600**	1	25/33	30/37	19/27	21/26	95/123	77

**600**	1.25	20/25	14/19	20/23	15/19	69/86	80

**600**	1.5	6/10	17/20	10/14	5/10	38/54	70

**800**	1	27/41	18/27	18/22	25/33	88/123	72

**800**	1.25	12/15	4/5	7/8	9/10	32/38	84

**800**	1.5	9/10	12/14	10/13	9/10	40/47	85

Electroporation was performed at combinations of three different resistances (400, 600, 800 Ω) and three different voltages (1.0, 1.25, and 1.5 kV) at 25 μF capacitance. The number of GFP expressing colonies and the total number of hygromycin-resistant colonies obtained from four different experiments at designated electroporation conditions are indicated in the table. Combined results of the four experiments are also presented.

**Figure 4:**
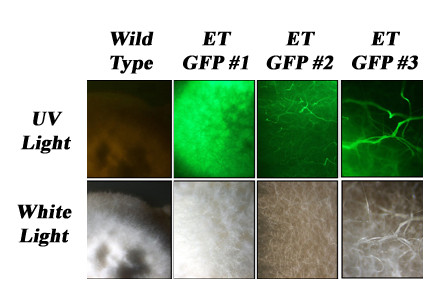
**Visualization of GFP-expressing *Epichloë typhina *transformants**. Fluorescent (top) and light (bottom) microscopic images of wild type and transformed *E. typhina*. A LEICA Fluorescent Dissecting Microscope MZFLIII with a PLANAPO 1.0X Lens was used to visualize GFP expression. Note: In order to visualize the untransformed control fungus shown above, low intensity white light was used in addition to the UV light.

Over 100 colonies were isolated and over 25 were chosen and stored long term. The percentage of hygromycin-resistant colonies expressing GFP was 70-85% and did not follow a specific pattern in relation to voltage or resistance applied (Table 1). GFP expression levels were variable and different colony morphologies were observed (Figure 4). Over 80% of the transformed lines continued to express GFP after successive transfers and storage. Fifteen randomly selected lines were verified as *E. typhina *by PCR using species specific primers (ACT1-F 5'-CCCCGCTCGGCTC-3' and ACT1-R 5'-GCCCAGGCCGAAAAATTA-3') to produce a 481 bp amplicon from the *actin 1 *locus which was sequenced [40].

### Southern analysis to determine copy number

**Figure 5:**
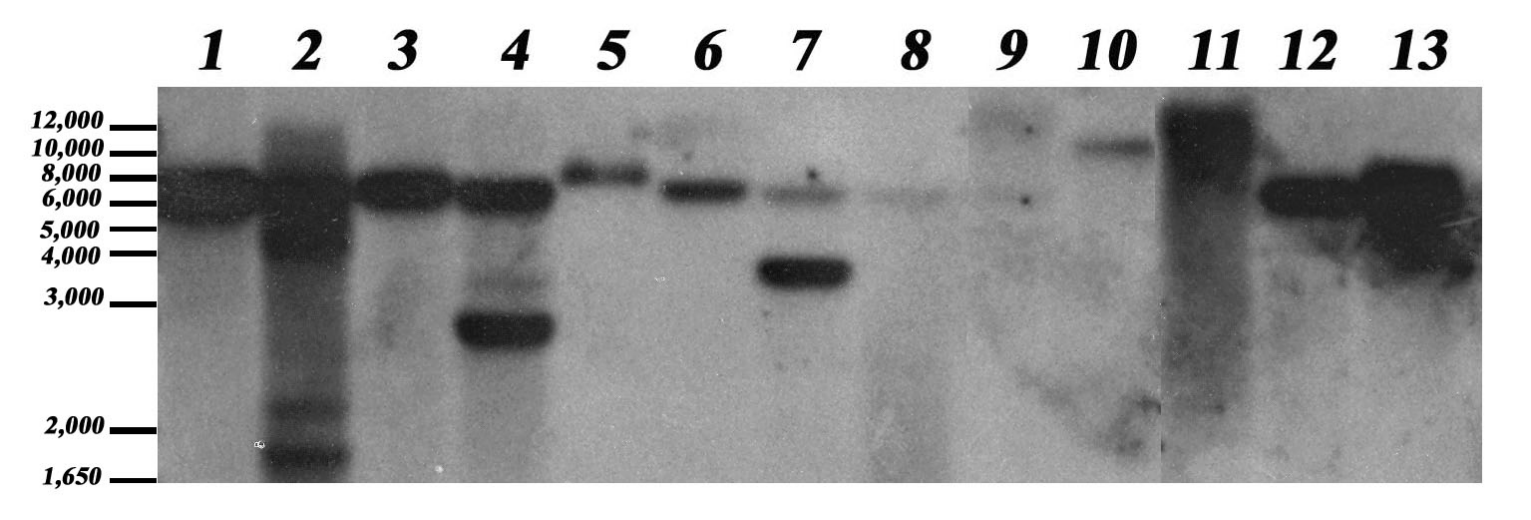
**Southern blot analysis of transformed *Epichloë typhina *lines**. Genomic DNA was digested with EcoRI and probed with a portion of the *GFP *gene (608 bp amplicon between nucleotide 64 and 671 of the *GFP *open reading frame) that was labelled with digoxigenin. The presence of a single band in most lanes indicates a single insertion event in those transformed lines. The different size of the bands indicates unique transformation events.

Southern analysis was performed to determine copy number for transformed fungal colonies. Approximately 70% of the lines tested contained single inserts and approximately 23% had two or three inserts (Figure 5). These 12 lines were found to be stable and were still expressing GFP one year after being established.

## Conclusions

An efficient method for the transformation of germinating conidia of *E. typhina *by electroporation was established. Two critical parameters for successful transformation were the use of a linearized plasmid and conidia that were collected from newly formed stromata. Transformed fungi were selected on 200 and 300 μg/mL hygromycin B. Over 80% of transformed lines continued to express GFP after successive transfers and storage. Fifteen lines were chosen and have been maintained for over 5 years and are still expressing GFP. We observed variable levels of GFP expression and some different colony morphologies. The selected isolates were verified as *E. typhina *by sequencing the PCR products obtained using species specific primers for the actin 1 locus. Southern analysis revealed the presence of a single insert in 70% of the lines tested. The GFP labelled *E. typhina *lines provide a valuable molecular tool to researchers studying conditions and mechanisms involved in the establishment of choke in orchardgrass. While the mode of infection for *E. typhina *in orchardgrass is uncertain, several researchers have successfully introduced *E. typhina *into perennial ryegrass and orchardgrass [5,41] however, it was not determined if the inoculated plants would ultimately manifest choke symptoms. It will be interesting to see if these methods will work to introduce the GFP expressing *E. typhina *into orchardgrass, and more importantly, if that will lead to the manifestation of choke disease in subsequent years. This would allow monitoring of the infection process under natural mechanisms of infection which would be quite different from artificial methods of inoculation.

## Abbreviations

GFP: Green Fluorescent Protein; CMMA: Corn Meal Malt Agar; NaOAc: Sodium Acetate; EtOH: Ethanol; μF: microfarad; RNase: ribonuclease; kV: kilovolts; Ω: Ohms

## Competing interests

The authors declare that they have no competing interests.

## Authors' contributions

JED, SCA and JCB conceived the study and participated in its design. SCA provided the fungal materials. JED and JCB carried out the transformation experiments and helped to draft the manuscript. RCM performed the Southern analysis and helped write the manuscript. All authors read and approved the final manuscript.

## Supplementary Material

Additional file 1 **Germination of collected conidia**. Isolated conidia were gently shaken in deionized water for 4 hours at room temperature. After 4 hours greater than 90% of conidia had germinated. Click here for file

Additional file 2 **Effect of electrical field strength on germinated conidia viability**. Electroporation was performed using the Bio-Rad Gene Pulser II with different voltages and resistances at 25 μF capacitance on 100 μL of suspended conidia in a 0.2 cm cuvette. The best electroporation conditions to use for transformation are those that result in 40-60% survival of the cells (between the green lines). Click here for file
