# Supplementary material for: Transformation of Epichloë typhina by electroporation of conidia
Source: BMC Res Notes. 2011 Mar 5;4:46. doi: 10.1186/1756-0500-4-46 (PMC3058031; doi:10.1186/1756-0500-4-46)
Supplement: Additional file 1 — Germination of collected conidia. Isolated conidia were gently shaken in deionized water for 4 hours at room temperature. After 4 hours greater than 90% of conidia had germinated. [file 1756-0500-4-46-S1.DOC]

## Supplementary Figure 1 - Germination of collected conidia

Isolated conidia were gently shaken in deionized water for 4 hours at room temperature. After 4 hours greater than 90% of conidia are germinated.
