# Supplementary material for: Transformation of Epichloë typhina by electroporation of conidia
Source: BMC Res Notes. 2011 Mar 5;4:46. doi: 10.1186/1756-0500-4-46 (PMC3058031; doi:10.1186/1756-0500-4-46)
Supplement: Additional file 2 — Effect of electrical field strength on germinated conidia viability. Electroporation was performed using the Bio-Rad Gene Pulser II with different voltages and resistances at 25 μF capacitance on 100 μL of suspended conidia in a 0.2 cm cuvette. The best electroporation conditions to use for transformation are those that result in 40-60% survival of the cells (between the green lines). [file 1756-0500-4-46-S2.DOC]

**0**

**10**

**20**

**30**

**40**

**50**

**60**

**70**

**80**

**90**

**100**

**1**

**1.25**

**1.5**

**1.75**

**2**

**0**

**10**

**20**

**30**

**40**

**50**

**60**

**70**

**80**

**90**

**100**

**1**

**1.25**

**1.5**

**1.75**

**2**

**400 Ω**

**600 Ω**

**800 Ω**

**Voltage (kV)**

**0**

**10**

**20**

**30**

**40**

**50**

**60**

**70**

**80**

**90**

**100**

**1**

**1.25**

**1.5**

**1.75**

**2**

**Percent survival**

## Supplementary Figure 2 - Effect of electrical field strength on germinated conidia viability

Electroporation was performed using the Bio-Rad Gene Pulser II with different voltages and resistances at 25 μF capacitance on 100 μL of suspended conidia in a 0.2 cm cuvette. The best electroporation conditions to use for transformation are those that result in 40-60% survival of the cells (between the green lines).
